# Supplementary material for: Modeling glioblastoma heterogeneity as a dynamic network of cell states
Source: Mol Syst Biol. 2021 Sep 16;17(9):e10105. doi: 10.15252/msb.202010105 (PMC8444284; doi:10.15252/msb.202010105)
Supplement: Supplementary file 5 — Source Data for Figure 3 [file MSB-17-e10105-s001.zip › Figure3A_sourcedata/GSEA_3065/nosigdb_state1.GseaPreranked.1622718409535/FELDMAN_2020_G1.html]

Details for gene set FELDMAN\_2020\_G1[GSEA]

|  || Dataset | state1 |
| Phenotype | NoPhenotypeAvailable |
| Upregulated in class | na\_pos |
| GeneSet | FELDMAN\_2020\_G1 |
| Enrichment Score (ES) | 0.50385326 |
| Normalized Enrichment Score (NES) | 1.9224055 |
| Nominal p-value | 0.0 |
| FDR q-value | 4.1415246E-4 |
| FWER p-Value | 0.007 |
Table: GSEA Results Summary

  

Fig 1: Enrichment plot: FELDMAN\_2020\_G1      
 Profile of the Running ES Score & Positions of GeneSet Members on the Rank Ordered List

  

| PROBE | GENE SYMBOL | GENE\_TITLE | RANK IN GENE LIST | RANK METRIC SCORE | RUNNING ES | CORE ENRICHMENT || 1 | SCG2 |  |  | 5 | 0.825 | 0.0318 | Yes |
| 2 | IGFBP3 |  |  | 15 | 0.664 | 0.0569 | Yes |
| 3 | UBE2S |  |  | 19 | 0.634 | 0.0814 | Yes |
| 4 | MAP3K7CL |  |  | 32 | 0.546 | 0.1016 | Yes |
| 5 | PTTG1 |  |  | 40 | 0.520 | 0.1212 | Yes |
| 6 | C12orf75 |  |  | 66 | 0.432 | 0.1356 | Yes |
| 7 | TUBB4B |  |  | 67 | 0.429 | 0.1524 | Yes |
| 8 | IGFBP5 |  |  | 88 | 0.385 | 0.1654 | Yes |
| 9 | TUBA1B |  |  | 119 | 0.347 | 0.1760 | Yes |
| 10 | UBE2C |  |  | 125 | 0.340 | 0.1888 | Yes |
| 11 | H2AFZ |  |  | 153 | 0.321 | 0.1986 | Yes |
| 12 | RANBP1 |  |  | 154 | 0.320 | 0.2111 | Yes |
| 13 | HMGN2 |  |  | 164 | 0.313 | 0.2225 | Yes |
| 14 | SNRPB |  |  | 172 | 0.310 | 0.2339 | Yes |
| 15 | CDKN3 |  |  | 178 | 0.307 | 0.2454 | Yes |
| 16 | RAN |  |  | 194 | 0.297 | 0.2555 | Yes |
| 17 | CKS2 |  |  | 195 | 0.297 | 0.2671 | Yes |
| 18 | BIRC5 |  |  | 202 | 0.295 | 0.2781 | Yes |
| 19 | CYCS |  |  | 203 | 0.294 | 0.2896 | Yes |
| 20 | DTYMK |  |  | 205 | 0.292 | 0.3009 | Yes |
| 21 | SIVA1 |  |  | 252 | 0.276 | 0.3070 | Yes |
| 22 | DEK |  |  | 296 | 0.261 | 0.3128 | Yes |
| 23 | CKS1B |  |  | 346 | 0.242 | 0.3172 | Yes |
| 24 | PTGES3 |  |  | 359 | 0.237 | 0.3253 | Yes |
| 25 | LSM3 |  |  | 361 | 0.237 | 0.3345 | Yes |
| 26 | PTMA |  |  | 366 | 0.236 | 0.3434 | Yes |
| 27 | PHF19 |  |  | 415 | 0.223 | 0.3472 | Yes |
| 28 | LSM5 |  |  | 448 | 0.216 | 0.3523 | Yes |
| 29 | MZT1 |  |  | 449 | 0.216 | 0.3608 | Yes |
| 30 | CDC20 |  |  | 469 | 0.212 | 0.3671 | Yes |
| 31 | TMEM106C |  |  | 478 | 0.211 | 0.3746 | Yes |
| 32 | H2AFV |  |  | 538 | 0.198 | 0.3763 | Yes |
| 33 | AURKA |  |  | 550 | 0.196 | 0.3828 | Yes |
| 34 | TPX2 |  |  | 556 | 0.194 | 0.3899 | Yes |
| 35 | CCNB1 |  |  | 583 | 0.189 | 0.3946 | Yes |
| 36 | CALM2 |  |  | 597 | 0.186 | 0.4006 | Yes |
| 37 | ANP32B |  |  | 622 | 0.183 | 0.4053 | Yes |
| 38 | PDLIM7 |  |  | 644 | 0.180 | 0.4102 | Yes |
| 39 | SNRPD1 |  |  | 647 | 0.179 | 0.4170 | Yes |
| 40 | CMC2 |  |  | 722 | 0.169 | 0.4160 | Yes |
| 41 | ILF2 |  |  | 729 | 0.167 | 0.4219 | Yes |
| 42 | GJB2 |  |  | 750 | 0.166 | 0.4264 | Yes |
| 43 | STMN1 |  |  | 763 | 0.163 | 0.4315 | Yes |
| 44 | CENPN |  |  | 764 | 0.163 | 0.4379 | Yes |
| 45 | CDCA2 |  |  | 859 | 0.150 | 0.4341 | Yes |
| 46 | CMTM7 |  |  | 868 | 0.148 | 0.4391 | Yes |
| 47 | HMGB3 |  |  | 907 | 0.144 | 0.4408 | Yes |
| 48 | C6orf15 |  |  | 921 | 0.143 | 0.4451 | Yes |
| 49 | ANLN |  |  | 941 | 0.141 | 0.4487 | Yes |
| 50 | CEP55 |  |  | 986 | 0.135 | 0.4494 | Yes |
| 51 | CDCA3 |  |  | 994 | 0.134 | 0.4540 | Yes |
| 52 | HMGB1 |  |  | 995 | 0.134 | 0.4592 | Yes |
| 53 | RPA3 |  |  | 1039 | 0.129 | 0.4599 | Yes |
| 54 | TK1 |  |  | 1056 | 0.127 | 0.4632 | Yes |
| 55 | HNRNPDL |  |  | 1086 | 0.124 | 0.4651 | Yes |
| 56 | TROAP |  |  | 1134 | 0.119 | 0.4649 | Yes |
| 57 | NUDT1 |  |  | 1144 | 0.118 | 0.4686 | Yes |
| 58 | DLG1 |  |  | 1217 | 0.111 | 0.4656 | Yes |
| 59 | CCNB2 |  |  | 1232 | 0.110 | 0.4684 | Yes |
| 60 | CENPU |  |  | 1249 | 0.108 | 0.4710 | Yes |
| 61 | DDX39A |  |  | 1255 | 0.108 | 0.4747 | Yes |
| 62 | CENPW |  |  | 1278 | 0.106 | 0.4766 | Yes |
| 63 | KIF4A |  |  | 1303 | 0.103 | 0.4782 | Yes |
| 64 | AHNAK2 |  |  | 1306 | 0.103 | 0.4820 | Yes |
| 65 | CKAP2L |  |  | 1359 | 0.099 | 0.4805 | Yes |
| 66 | MAD2L1 |  |  | 1394 | 0.095 | 0.4808 | Yes |
| 67 | PLK1 |  |  | 1403 | 0.095 | 0.4837 | Yes |
| 68 | DHFR |  |  | 1413 | 0.094 | 0.4864 | Yes |
| 69 | CTGF |  |  | 1416 | 0.094 | 0.4899 | Yes |
| 70 | UBE2T |  |  | 1440 | 0.093 | 0.4912 | Yes |
| 71 | DEPDC1 |  |  | 1478 | 0.090 | 0.4909 | Yes |
| 72 | H2AFX |  |  | 1479 | 0.090 | 0.4945 | Yes |
| 73 | TYMS |  |  | 1501 | 0.089 | 0.4958 | Yes |
| 74 | NUCKS1 |  |  | 1540 | 0.087 | 0.4953 | Yes |
| 75 | CENPE |  |  | 1545 | 0.086 | 0.4983 | Yes |
| 76 | HMGB2 |  |  | 1570 | 0.085 | 0.4991 | Yes |
| 77 | DNAJC9 |  |  | 1594 | 0.083 | 0.5000 | Yes |
| 78 | HIST1H1E |  |  | 1608 | 0.082 | 0.5019 | Yes |
| 79 | KIF23 |  |  | 1638 | 0.080 | 0.5020 | Yes |
| 80 | PBK |  |  | 1651 | 0.079 | 0.5039 | Yes |
| 81 | ANP32E |  |  | 1706 | 0.075 | 0.5012 | No |
| 82 | NUSAP1 |  |  | 1736 | 0.073 | 0.5011 | No |
| 83 | CENPA |  |  | 1788 | 0.070 | 0.4986 | No |
| 84 | PRC1 |  |  | 1794 | 0.070 | 0.5009 | No |
| 85 | CCNA2 |  |  | 1835 | 0.068 | 0.4994 | No |
| 86 | EXOSC8 |  |  | 1851 | 0.067 | 0.5005 | No |
| 87 | KIF22 |  |  | 1858 | 0.067 | 0.5025 | No |
| 88 | USP1 |  |  | 1921 | 0.064 | 0.4987 | No |
| 89 | ZWINT |  |  | 1923 | 0.064 | 0.5011 | No |
| 90 | TPST2 |  |  | 1932 | 0.063 | 0.5027 | No |
| 91 | DLGAP5 |  |  | 2028 | 0.058 | 0.4952 | No |
| 92 | MIS18A |  |  | 2094 | 0.055 | 0.4907 | No |
| 93 | GMNN |  |  | 2097 | 0.055 | 0.4927 | No |
| 94 | CYR61 |  |  | 2172 | 0.052 | 0.4871 | No |
| 95 | CENPH |  |  | 2215 | 0.051 | 0.4848 | No |
| 96 | SMC4 |  |  | 2308 | 0.047 | 0.4771 | No |
| 97 | CENPM |  |  | 2348 | 0.045 | 0.4749 | No |
| 98 | FEN1 |  |  | 2420 | 0.043 | 0.4693 | No |
| 99 | KNSTRN |  |  | 2515 | 0.039 | 0.4611 | No |
| 100 | HSP90AA1 |  |  | 2562 | 0.038 | 0.4579 | No |
| 101 | GINS2 |  |  | 2567 | 0.038 | 0.4590 | No |
| 102 | DNMT1 |  |  | 2576 | 0.037 | 0.4596 | No |
| 103 | GTSE1 |  |  | 2703 | 0.033 | 0.4479 | No |
| 104 | HIST1H1C |  |  | 2712 | 0.033 | 0.4484 | No |
| 105 | DBF4 |  |  | 2751 | 0.031 | 0.4457 | No |
| 106 | MKI67 |  |  | 2760 | 0.031 | 0.4461 | No |
| 107 | CKAP2 |  |  | 2763 | 0.031 | 0.4471 | No |
| 108 | FBXO5 |  |  | 2870 | 0.028 | 0.4373 | No |
| 109 | DUT |  |  | 3005 | 0.025 | 0.4245 | No |
| 110 | TMPO |  |  | 3093 | 0.023 | 0.4165 | No |
| 111 | VRK1 |  |  | 3288 | 0.018 | 0.3973 | No |
| 112 | CDK1 |  |  | 3396 | 0.016 | 0.3869 | No |
| 113 | PCNA |  |  | 3540 | 0.013 | 0.3727 | No |
| 114 | NCAPG |  |  | 4261 | -0.000 | 0.2987 | No |
| 115 | NUF2 |  |  | 4342 | -0.002 | 0.2905 | No |
| 116 | SRSF7 |  |  | 4413 | -0.003 | 0.2835 | No |
| 117 | CENPK |  |  | 4697 | -0.007 | 0.2547 | No |
| 118 | SPC25 |  |  | 4740 | -0.008 | 0.2507 | No |
| 119 | AURKB |  |  | 4764 | -0.008 | 0.2486 | No |
| 120 | BUB3 |  |  | 4828 | -0.010 | 0.2425 | No |
| 121 | RAD21 |  |  | 4916 | -0.011 | 0.2340 | No |
| 122 | HIST1H1B |  |  | 5125 | -0.014 | 0.2132 | No |
| 123 | KIF20B |  |  | 5169 | -0.015 | 0.2094 | No |
| 124 | CCDC34 |  |  | 5298 | -0.017 | 0.1969 | No |
| 125 | KPNA2 |  |  | 5461 | -0.020 | 0.1810 | No |
| 126 | PSIP1 |  |  | 5794 | -0.025 | 0.1478 | No |
| 127 | CLSPN |  |  | 5848 | -0.026 | 0.1434 | No |
| 128 | HNRNPA2B1 |  |  | 6142 | -0.032 | 0.1145 | No |
| 129 | RRM2 |  |  | 6214 | -0.033 | 0.1085 | No |
| 130 | NDC80 |  |  | 6240 | -0.034 | 0.1073 | No |
| 131 | RNASEH2A |  |  | 6891 | -0.047 | 0.0423 | No |
| 132 | CENPF |  |  | 6902 | -0.047 | 0.0431 | No |
| 133 | LMNB1 |  |  | 7166 | -0.055 | 0.0182 | No |
| 134 | MCM7 |  |  | 7397 | -0.061 | -0.0030 | No |
| 135 | RRM1 |  |  | 7556 | -0.065 | -0.0167 | No |
| 136 | TOP2A |  |  | 7678 | -0.069 | -0.0265 | No |
| 137 | ASPM |  |  | 7821 | -0.074 | -0.0382 | No |
| 138 | HAT1 |  |  | 7958 | -0.080 | -0.0490 | No |
| 139 | ESCO2 |  |  | 8566 | -0.109 | -0.1071 | No |
| 140 | GGH |  |  | 8643 | -0.114 | -0.1105 | No |
| 141 | SMC2 |  |  | 8721 | -0.119 | -0.1137 | No |
| 142 | HIST1H4C |  |  | 8820 | -0.126 | -0.1189 | No |
| 143 | ORC6 |  |  | 8888 | -0.133 | -0.1205 | No |
| 144 | ATAD2 |  |  | 8948 | -0.138 | -0.1212 | No |
| 145 | ARL6IP1 |  |  | 9038 | -0.146 | -0.1246 | No |
| 146 | PLP1 |  |  | 9702 | -0.314 | -0.1805 | No |
| 147 | MIAT |  |  | 9732 | -0.341 | -0.1701 | No |
| 148 | S100B |  |  | 9822 | -0.507 | -0.1593 | No |
| 149 | PRCP |  |  | 9855 | -0.712 | -0.1347 | No |
| 150 | MALAT1 |  |  | 9868 | -0.912 | -0.1002 | No |
| 151 | PTN |  |  | 9874 | -1.107 | -0.0573 | No |
| 152 | SCRG1 |  |  | 9879 | -1.473 | -0.0000 | No |
Table: GSEA details [plain text format]

  

Fig 2: FELDMAN\_2020\_G1: Random ES distribution      
 Gene set null distribution of ES for **FELDMAN\_2020\_G1**
